# Posterior scoliosis correction for adolescent idiopathic scoliosis using side-opening pedicle screw-rod system utilizing the axial translation technique

**DOI:** 10.4103/0019-5413.58605

**Published:** 2010

**Authors:** Saumyajit Basu, Sreeramalingam Rathinavelu, Prashant Baid

**Affiliations:** Park Clinic, Neurosciences Division, Kolkata, India

**Keywords:** Adolescent idiopathic scoliosis, axial translation technique, side opening pedicle screws

## Abstract

**Background:**

Though adequate literature is present depicting the results of pedicle screw-rod instrumentation using top loading systems for correction of adolescent idiopathic scoliosis (AIS), using the rod rotation technique, few published data is available regarding side loading systems used for a similar purpose. We report a retrospective study of a cohort of patients with strict inclusion criteria who underwent surgical correction of AIS with side-opening pedicle screw-rod posterior instrumentation using the axial translation technique of curve correction to assess the efficacy of side opening system for scoliosis correction with regards to patient satisfaction, Cobb's angle correction and spinal balance.

**Materials and Methods:**

Clinical and radiological outcomes were measured in 14 consecutive patients (3 males, 11 females) with an average age of 14.0 years (range 9 to 23 years). They were followed up for an average period of 13.0 months (range – 2.2 to 28.5). All patients underwent posterior instrumentation only with pedicle screws used as anchor points. Hybrid constructs using hooks/wires or curves requiring anterior release were excluded from the study. All levels were not instrumented – more screws were put on the concavity and in the peri-apical region. Radiological evaluation was done by whole spine standing AP, lateral radiograms preoperatively and 1, 3, 6 and12 months after surgery. Cobb's angles were measured and the spinal balance was noted. Clinical evaluation was done by SRS questionnaire. The complications were documented.

**Results:**

The mean preoperative Cobb's angle was 58.35° (range – 44 to 72°), which came down postoperatively to 23.45° (range – 10 to 38°) signifying a mean correction of 59.57% (range – 26.92 to 76.17%). Clinical outcomes were evaluated using the SRS – 30 questionnaires. The values of mean pre- and postoperative scores are 3.68 and 4.18, showing an improvement of 0.5 points. Other than one patient of superficial wound infection, which healed with antibiotics, there was no major complication. No patient had neurological deterioration.

**Conclusion:**

Side-opening spinal instrumentation systems, using the axial translation technique, achieved good clinical and radiological outcome for patients of AIS.

## INTRODUCTION

In the past few decades we have seen revolutionary changes in the treatment of Adolescent Idiopathic Scoliosis (AIS). Although we still follow the osseous fusion introduced by Harrington^1^ in early 1960s, we have come a long way from the concept of deformity correction by ‘distraction’. The segmental fixation technique by sublaminar wiring, first introduced by Luque^2^ and later modified by replacing wires with hooks/screws added the advantage of segmental compression/distraction. The concept of rod rotation as a mechanism of scoliosis correction was introduced later by Cotrel and Dubousset.^3^ Experience with Cotrel and Dubousset (CD) system showed that spine could become unbalanced above and below the construct. To overcome this disadvantage, universal spine system (USS) was designed,^4^ in which the cranial and caudal ends of the construct are fixed to rods to create a frame. Anchor points placed in intervening segments are translated into the frame – the axial translation technique. Active derotation in each of the segments can also be applied through this system. This instrumentation also allows restoration of the thoracic kyphosis and realignment of thoracic torsion by lifting the concavity out of the chest and reducing the convex rib deformity. This is a retrospective study of a cohort of patients with strict inclusion criteria who underwent surgical correction of AIS with side-opening screw-rod posterior instrumentation using the axial translation technique to assess the efficacy of side opening system for scoliosis correction with regards to patient satisfaction, Cobb's angle correction and spinal balance.

## MATERIALS AND METHODS

Fourteen consecutive patients who underwent posterior correction of scoliosis with all pedicle screw construct (USS) – manufactured by Synthes – by the axial translation technique between 2006 and 2008 were included in the study. The indication for surgery in these cases was Cobb's angle of 40° or more but less than 90° with significant chest wall deformity and coronal decompensation.

**Figure 1:**
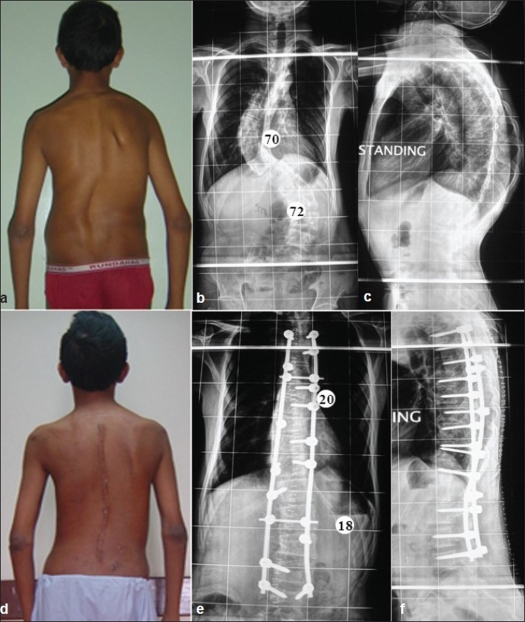
Clinical picture (a) and whole spine anteroposterior (b) and lateral (c) radiographs showing typical King Type I curve with Cobb's angle of 70° (thoracic) and 72° (Lumbar). Follow up clinical picture (d) and radiograph anteroposterior (e) and lateral (f) showing good correction and reduction of Cobb's angle to 20° (thoracic) and 18° (Lumbar)

Medical case sheets and investigations including X rays and MRI of the patients were studied retrospectively. The patient details are shown in Table 1. All the patients were available for follow-up. The average follow-up period is 13 months (range 10 to 24 months). There were 3 males and 11 females. The average age was 14 years (range 9 to 23 years). King and Moe classification system^5^ was followed. There were four patients with Type 1 curve [Figure 1], two with Type 2 curve [Figure 2a and b], five patients with Type 3 curve [Figure 3] and three patients with Type 4 curve [Figure 4], [Table 1]. There were no Type V patients in our series.

**Figure 2a:**
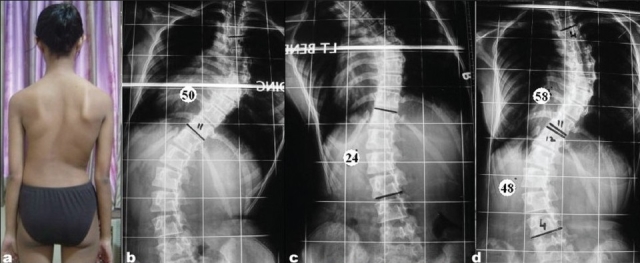
(a) Preoperative clinical picture (a) and whole spine standing anteroposterior (b), right and left bending X-rays (c,d) of typical King type II curve. Thoracic curve (T4-T11) is more and less flexible than lumbar with Cobb's angle 58° in thoracic and 48° in Lumbar

**Figure 2b:**
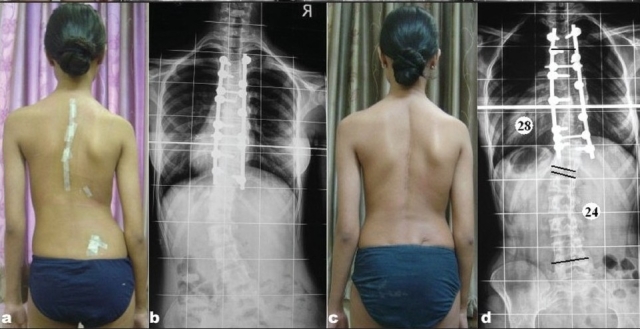
Immediate postoperative clinical picture (a) and anteroposterior radiograph (b) depicting slight decompensation. 6 months follow up clinical picture (c) and radiograph (d) showing restored coronal spinal balance with final Cobb's angles; thoracic 28°, Lumbar 24°

**Figure 3:**
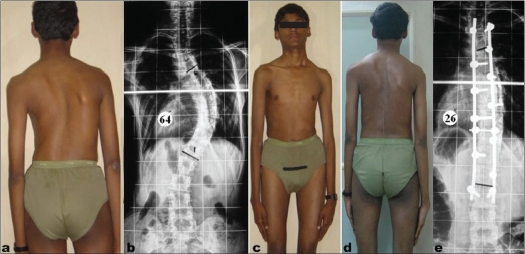
Clinical photograph (a) and anteroposterior radiograph (b) showing typical King type III (T6-L1) curve in Marfan syndrome as the hands have crossed the mid thigh (c). Cobb's angle is 64°. Follow up clinical (d) and radiographic (e) shows correction with Cobb's angle that has been reduced to 26°

**Figure 4:**
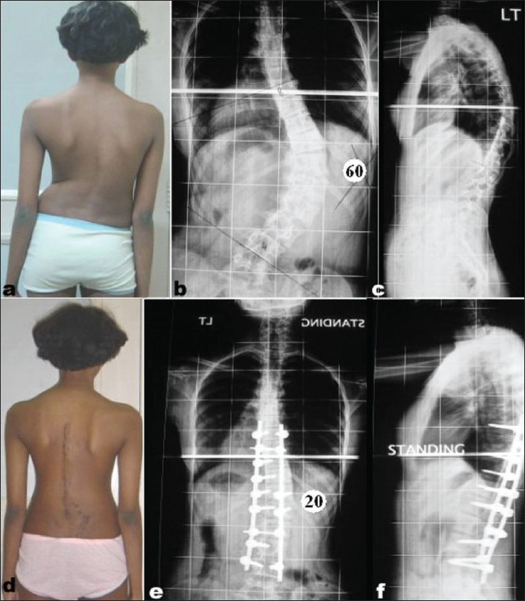
Clinical photograph (a) and anteroposterior (b)and lateral (c) radiograph showing typical King type IV curve with L4 tilted to the convexity of the curve with preoperative Cobb's angle of 60°. (ii) Follow up clinical picture (d) and radiographs, anteroposterior (e) and lateral (f) showing good correction. Cobb's angle has been reduced to 20°

**Table 1:** Pre/postoperative and final follow-up Cobb's angles and the percentage correction achieved

Patient	Curve type (King/Moe)	Pre-op Cobb	Immediate post-op Cobb	Final FU Cobb	% correction	UIV	LIV
11	I	52 (T)/62 (L)	34 (T)/35 (L)	38 (T)/38 (L)	33	T4	L4
10	I	48 (T)/64 (L)	18 (T)/22 (L)	20 (T)/24 (L)	61	T4	L4
9	I	50 (T)/48 (L)	19 (T)/8 (L)	22 (T)/10 (L)	68	T3	L4
12	I	70 (T)/72 (L)	18 (T)/16 (L)	20 (T)/18 (L)	73	T4	L4
7	II	64 (T)/44 (L)	28 (T)/14 (L)	30 (T)/(16L)	59	T2	T12
8	II	50 (T)/48 (L)	27 (T)/14 (L)	30 (T)/(16L)	54	T2	L2
3	III	45	13	15	67	T5	L2
1	III	72	28	32	56	T3	L3
6	III	64	23	26	59	T4	L3
13	III	62	18	20	68	T3	L3
14	III	68	24	26	62	T2	L1
2	IV	60	18	20	67	T8	L4
4	IV	60	25	28	53	T9	L4
5	IV	64	18	20	69	T3	L4
Average		58.35	20.01	23.45	59.57		

UIV – Upper instrumented vertebra; LIV – Lower instrumented vertebra

### Operative procedure

**Figure 5:**
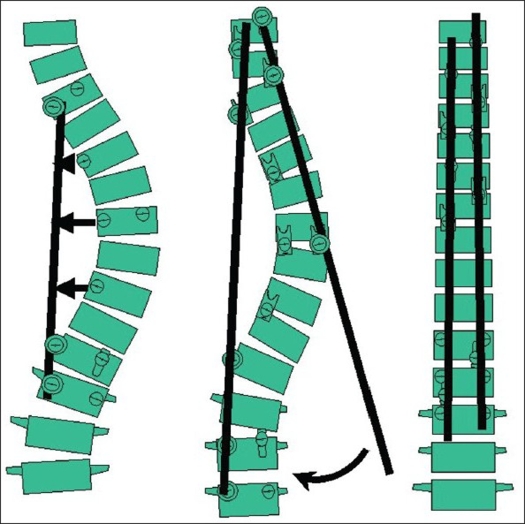
Axial translation technique of scoliosis correction - Concave rod insertion at the ends followed by convex rod insertion from above downwards followed by approximation of concave rod to the apical anchors

Good preoperative planning is a must before the surgery. Preoperative assessment of the type of curve is important to choose the levels of fusion. The levels at which the screws were to be applied, the upper and the lower level of instrumentation, the technique to be used for correction and anticipated complications were all taken into consideration at the time of planning. Patients were positioned prone under general anesthesia. Exposure of the desired level was achieved with a subperiosteal approach. Side-opening mono-axial pedicle screw fixation was carried out according to the plan charted out preoperatively. All levels were not instrumented – more screws were put on the concavity and in the periapical region. Facet release and costo-transverse release was carried out. The concave rod was first applied to the upper and lower screws and one of them was tightened fully, while in the other, the rod was captured but not fully tightened. This enabled the rod to slide as and when curve correction occured. Then the convex rod was applied to the upper level of the construct and gradual capturing of the subsequent screws was done from the top to the bottom, thus providing axial translation of the spine. The rest of the anchor points on the concave side were now gradually captured with the apical ones being captured last. Complex reduction forceps, provided in the instrumentation was highly useful in this technique. All the screws in the concave and the convex sides having been captured, they were fully tightened [Figure 5]. Wake up test was done at this point to confirm that the patient is neurologically intact. Bone grafting from local bone iliac crest graft and beta tricalcium phosphate was done to ensure adequate fusion. Wound was closed in layers after putting a closed suction drain. Depending on pain relief, the patient was mobilized with a brace by the third to the fifth day and discharged after suture removal on the tenth day. They usually were allowed to join school after one month and physical training was allowed after 3 months. Contact sports are allowed at least after 6 to 9 months.

The clinical assessment of all these patients were carried out preoperatively and 1, 3, 6 and 12 months after surgery. They were given the SRS 30 Outcomes Questionnaire. The SRS 30 Outcomes Questionnaire contains 30 questions, which spans over five domains – function, pain, self-image, mental health and satisfaction with management. Each domain is scored from 1 (worst) to 5 (best), and the results are obtained as the mean of each domain. The surgery details, the operative time, blood loss, perioperative course and the complications were obtained from the case notes.

### Radiological evaluation

All the patients underwent whole spine standing antero-posterior and lateral views along with supine right and left bending antero-posterior views and traction films in the preoperative period. Whole spine standing antero-posterior and lateral views were taken after removal of the drains in the postoperative period, at 6 months, at 12 months and yearly from then on. Cobb's angle measurements, Thoracic Sagittal Profile – T5–T12, C7 plumb line, apical vertebral translation (standing radiographs) and apical vertebral rotation (Nash–Moe grading) were noted in all these patients. The upper level of instrumentation, lower level of instrumentation and instrumented levels were recorded. In the postoperative radiograph special attention was also given for signs of fusion, instrumentation failure and pseudoarthrosis.

## RESULTS

All the 14 patients were available for follow-up (minimum 10 months, maximum 24 months, and average 13 months). The mean pre- and postoperative SRS scores are shown in Table 2. The maximum improvement occurred in domains of self image and satisfaction. The scores increased from an average of 3.68 to 4.08 at immediate postoperative, which implies an improvement from 73.6 to 81.6% -an average improvement of 8%, which went on to 10% at final follow-up with mean score 4.18.

**Table 2:** Scoliosis Research Society scores for patient satisfaction after surgery

SRS scores variables	Preop	Immediate postop	Final FU
Function	3.5	3.5	3.5
Pain	4.1	4.2	4.4
Image	2.8	4.1	4.2
Mental health	4.3	4.3	4.3
Satisfaction	3.7	4.3	4.5
Average improvement	3.68 (73.6%)	4.08 (81.6%)	4.18 (83.6%)

SRS = Scoliosis research society

### Radiological outcomes

The measurements were made from the preoperative, immediate postoperative and the last follow-up radiographs. The measurements show a mean preoperative Cobb's angle of 58.35° (44 to 72°) and the final follow-up mean Cobb's angle as 23.45° (10 to 38°). There was an improvement of 39.4°, i.e., 59.57% (26.92 to 76.17%). The details of radiographic measurements are shown in Table 1. There was a correction loss of 2 to 4° in the final follow-up radiographs.

The measurement of apical vertebral translation (AVT) showed that from a preoperative average of 23.61 mm, it decreased to 7.89 mm in the postoperative, which means a correction of 67.28% was achieved. We were able to achieve a correction of 61.53% in apical vertebral rotation (AVR). The preoperative and the postoperative Nash and Moe grades were 2.64 and 0.99, respectively. Table 3 shows the details.

**Table 3:** Shows the mean and standard (SD) of various parameters

Parameter	Mean	SD
AVR		
Preop	2.64	0.56
Postop	1.03	0.27
Final follow-up	0.99	0.24
AVT		
Preop	23.61	7.9
Postop	8.15	3.72
Final follow-up	7.89	3.54
C7 plumbline		
Preop	−14.09	16.01
Postop	−16.41	30.43
Final follow-up	−8.38	14.82
Thoracic sagittal profile – T5 to T12		
Preop	24.38	12.12
Postop	11.13	5.78
Final follow-up	12.17	6.55
SRS 30 scores		
Preop	3.68	0.58
Postop	4.08	0.33
Final followup	4.18	0.39
Cobb's angle measurements		
Preop	58.35	8.7
Postop	20.01	5.71
Final followup	23.45	6.28

AVR = Apical vertebral rotation, AVT = Apical vertebral translation

### Complications

We had one case of superficial infection, which healed, subsequently with regular dressings and antibiotics. There was no neurological deterioration in any of our cases. No long-term complications were noted. There was no evidence of pseudoarthrosis or instrumentation failure in the last follow-up.

## DISCUSSION

The quest for the better treatment for AIS will go on. The SRS 30 outcomes questionnaire is a very useful and validated tool in clinically assessing the patients with adolescent idiopathic scoliosis.^6^ It includes five domains – function, pain, self-image, mental health and satisfaction. In our study we saw major changes in the self-image and satisfaction domains, whereas the other three domains did not show much change in the postoperative period. Most of the patients felt that their new look is much better than what they were before. The improvement of SRS scores in our series is about 10%, at last followup which is near to the 7.1% quoted in the series of Lehmann and Lenke.^7^

A correction of 43.2% was shown on 22 patients with right thoracic scoliosis who were operated with CD system alone,^8^ which is a vast improvement over the Harrington era. While there remains little doubt that correction of Cobb's angle is significantly increased in the segmental systems, an excellent comparative analysis of pedicle screw versus hybrid instrumentation (using screws/hooks) was published by Kim and Lenke.^9^ They showed average major curve correction was 70% in the screw group and 56% in the hybrid group (*P* = 0.001). At two-year follow-up, major curve correction was 65 and 46%, respectively (*P* < 0.001). However the efficacy of pedicle screws at all levels needs to be validated. In our series, we have not used any hooks but have not used pedicle screws at all levels. More screws were used in the concavity and in the periapical region. The curve correction nearly matches those found by other authors.

Lonner and Boachie *et al.,*^10^ and Ma *et al.,*^11^ show an average improvement of 69 and 72.5%, respectively by using all pedicle screw constructs while Lehman and Lenke^7^ showed an improvement of 72.1% in their three-year follow-up results. Table 4 shows the comparison between a few studies with respect to correction of the Cobb's angle in the postoperative period.

**Table 4:** Cobb's angle correction achieved by various authors in different techniques of scoliosis correction

	Preoperative Cobb	Postoperative Cobb	% correction
Ma *et al.*^11^	62	18	72.5
Lenke *et al.*^7^	59.2	16.8	72.1
Suk *et al.*^12^	55	12	79.6
Vallespir *et al.*^13^	61	16	73
Boachie *et al.*^10^	56	14	69

There remain two other recent techniques of curve correction, namely the direct vertebral rotation and vertebral coplanar alignment. Lee and Suk *et al*.^12^ have described a correction of 79.6% in thoracic and 80.5% (39 to 7°) in lumbar curves using their technique of direct vertebral rotation. In this technique, pedicle screws are put in all the vertebrae at the concavity and a contoured rod is fitted into them followed by standard counter-clockwise derotation technique. In addition, vertical extenders are attached to the few apical screws and each of them is rotated clockwise before final tightening thus achieving direct derotation of the apical vertebrae. The convex screws (one every 2 or 3 vertebrae) are then locked onto a contoured rod and the rod is delivered in situ.

Vellespir *et al.*^13^ has shown a curve correction of 73% in their thoracic curves and 70% in thoracolumbar curves with the technique of vertebral coplanar alignment. The latter technique involves vertical slotted tubes attached to the convex screws and longitudinal rod is put in gradually to the convex side so that the rotated vertebrae all come in one plane. The concave rod is then inserted. No universal agreement however exists on the superiority of one over the other, nor are there specific curve patterns, which are suitable for one technique, rather than the other.

In our study, we were able to achieve a correction of 62.6% in thoracolumbar curves, 51% in thoracic curves and 64.3% in lumbar curves, which agreeably falls quite short of the correction achieved with these two recently described techniques.

The goals of vertebral derotation are to achieve true three-dimensional correction of the spinal deformity and reverse the torsional asymmetry induced by scoliosis. In our study, the type 3 and type 4 curves had a preoperative Nash–Moe grading of 2.8 and 3.33, which decreased to 0.8 and 1.33, respectively. In types 1 and 2 curves, preoperative grading was 2.43 and 2, which decreased to 0.86 and 1, respectively. Lehman *et al.*^7^ has shown an improvement of 2.0 to 1.1 for proximal thoracic curves and 1.6 to 1.1 for thoracolumbar/lumbar curves. Analyzing the data show that thoracic curves have undergone better derotation than the long thoracolumbar curves.

As far as the controversy on mono and polyaxial screws for correction are concerned, Lonner and Boachie *et al.*^10^ have concluded in their study that similar coronal and sagittal plane correction is achieved in thoracic adolescent idiopathic scoliosis with these constructs. Mono screw constructs have shown improved correction of clinical rib hump deformity when compared with poly screw constructs. However as there is no option of polyaxial screws in the system. We used USS-1; hence this issue does not arise. Literature comparing data of side opening systems with top opening systems for AIS is sparse. In our study, we have also not attempted to do a comparison between these two groups.

Scoliosis correction surgeries carry high probability of screw malpositioning than other surgeries involving pedicle screw insertion. Di Silvestre *et al.*^14^ in their study on complications of thoracic pedicle screws in the treatment of scoliosis show that although 18 screws out of 311 (i.e. 1.7%) were misplaced; only one patient developed symptom. Kuklo and Lenke *et al.*^15^ could show an accuracy of 96.3% with thoracic pedicle screws after reviewing postoperative CT scans in 20 patients with Cobb's angle of at least 90°. We did not have a single clinical problem due to screw malposition.

## CONCLUSION

Side-opening spinal instrumentation systems, using the axial translation technique, achieved good clinical and radiological outcome for patients of Adolescent Idiopathic Scoliosis (AIS).
